# PrEP Uptake and Utilisation Among Adolescent Girls and Young Women in Sub-Saharan Africa: A Scoping Review

**DOI:** 10.1007/s10461-025-04656-4

**Published:** 2025-02-28

**Authors:** Jenny Chen-Charles, Dvora Joseph Davey, Elona Toska, Janet Seeley, Linda-Gail Bekker

**Affiliations:** 1 Desmond Tutu HIV Centre, Department of Medicine, University of Cape Town, Cape Town, South Africa; 2 Department of Social Policy and Intervention, University of Oxford, Oxford, UK; 3 Department of Epidemiology and Biostatistics, School of Public Health, University of Cape Town, Cape Town, South Africa; 4 Division of Infectious Diseases, Geffen School of Medicine, University of California, Los Angeles, CA USA; 5 Centre for Social Science Research, University of Cape Town, Rondebosch, South Africa; 6 Africa Health Research Institute, Durban, KwaZulu-Natal South Africa; 7 School of Nursing and Public Health, College of Health Sciences, University of KwaZulu-Natal, Durban, South Africa; 8 Department of Global Health and Development, London School of Hygiene and Tropical Medicine, London, UK

**Keywords:** PrEP, Adolescent girls and young women (AGYW), Service delivery, Sub-Saharan Africa, Facilitators to PrEP, Barriers to PrEP, HIV prevention

## Abstract

Adolescent girls and young women (AGYW) in sub-Saharan Africa (SSA) are disproportionately affected by HIV. Despite the effectiveness of oral pre-exposure prophylaxis (PrEP) in preventing HIV, uptake and effective utilisation among AGYW remain suboptimal. This scoping review maps research on PrEP delivery outside clinical trials to AGYW in SSA. Quantitative and qualitative data were extracted from 58 studies on the facilitators and barriers to PrEP uptake and utilisation (including initiation, persistence, and adherence), and recommendations for effective PrEP delivery from AGYW and PrEP providers. Only studies on oral PrEP met the inclusion criteria. Facilitators of effective PrEP utilisation included social support with strong familial and peer networks positively influencing PrEP adherence and persistence. Healthcare provider interactions were pivotal in promoting PrEP uptake through dissemination of accurate information and ongoing support. Studies reported consistent barriers to PrEP uptake and utilisation including anticipated or experienced stigma, pill burden, and side effects. Addressing identified barriers and leveraging facilitators can enhance future effectiveness for PrEP delivery. There is a lack of strategies to support AGYW in long-term persistence and engagement with PrEP services. Our findings emphasise the urgent need for people-centred and localised, context-specific strategies to improve PrEP delivery among AGYW in SSA. Effective PrEP delivery strategies should include differentiated service delivery models, innovative approaches such as digital health, and integration with existing services such as antenatal care for pregnant and breastfeeding AGYW. More data is needed for PrEP delivery among AGYW across the region, including other PrEP modalities as they roll out.

**Supplementary Information:**

The online version contains supplementary material available at 10.1007/s10461-025-04656-4.

## Background

Globally, 3800 adolescent girls and young women (AGYW) acquired HIV every week in 2023, with 76% of those infections occurring in sub-Saharan Africa (SSA) [1]. In 2023, 198,000 AGYW (15–24 years old) were reported to have newly acquired HIV in SSA and were three times more likely to acquire HIV than their male counterparts [1]. This disproportionate burden of HIV among AGYW in the region may be due to a variety of biological, social, and structural factors, including gender-based violence, limited access to health services—including sexual and reproductive health (SRH) services, and socioeconomic vulnerabilities [2–4]. These statistics highlight the urgent need for comprehensive prevention programs tailored for AGYW.

Effective daily oral pre-exposure prophylaxis (PrEP) use can provide robust protection against HIV acquisition across all populations, reducing the chances of HIV acquisition to almost zero [5]. In 2015, WHO recommended PrEP for AGYW in high HIV burden areas, expanding in 2021 to include the dapivirine vaginal ring and in 2022 the Cabotegravir Long-Acting (CAB-LA) injections for individuals at risk of exposure [6]. More recently, the twice-yearly Lenacapavir injections have shown high efficacy in clinical trials, with no reported HIV infections among women in the PURPOSE-1 study [7]. However, the effectiveness of these methods relies on uptake and effective utilisation (uninterrupted use prior to time of exposure) as PrEP is a user-controlled HIV prevention method [8]. Women, including AGYW, in Africa have particularly reported challenges with PrEP adherence and persistence [9–13].

By 2024, approximately 6.7 million individuals globally had at least one use of oral PrEP administration, with a large contribution from South Africa, having surpassed 1.3 million oral PrEP initiations [14, 15]. Additionally, 144 nations have incorporated the WHO’s guidelines on oral PrEP into their national policies, with another 14 countries planning to implement these recommendations within the next two years [16]. However, effective PrEP uptake and utilisation is far from reaching the 2025 target, and access in low- and middle-income countries is still limited [1, 17]. Inequality in access is also clear within countries that have adopted the WHO PrEP recommendations and have widespread PrEP availability [18]. Many low- and middle-income countries rely on international donor-funded programmes to provide access to services and resources, suggesting significant variability in PrEP access both between and within countries [19, 20].

Given this context, we aimed to review existing publications around PrEP delivery among AGYW in SSA to identify barriers, facilitators, and recommendations for effective PrEP uptake and utilisation among AGYW in SSA. By doing so, we seek to inform tailored and effective interventions to improve PrEP uptake and adherence in this high-incidence population.

## Methods

A scoping review was conducted to assess the size and scope of existing literature on PrEP uptake and utilisation among AGYW in SSA, given the broad and varied nature of the available studies [21]. The PRISMA framework for conducting scoping reviews was used to guide this review (see Table 1 for the population, concept and context, and see Supplementary Appendix 1 for detailed steps taken) [22]. This review included both quantitative and qualitative evidence, therefore mixed methods research syntheses following Joanna Briggs Institute (JBI)’s convergent segregated approach was used to synthesise and present the findings. Quantitative and qualitative findings, including from mixed methods studies, were first analysed separately followed by convergence of the findings (see Supplementary Appendix 2) [23].

**Table 1:** Population, concept, and context for the review

*Population*
The population included in this study were adolescent girls and young women (ages 10–24 years)
*Concept*
Real-world PrEP roll-out and delivery
*Context*
This review considered studies from sub-Saharan Africa

### Data Sources and Search Strategy

We systematically searched relevant peer-reviewed literature using search terms related to PrEP, AGYW, delivery and implementation, and names of all SSA countries (see Supplementary Appendix 3 for the search terms). Databases searched include PubMed and Ovid (including Embase, MEDLINE, Scopus, Global Health, PsycInfo). Publications were limited to records that were published after 2012, when PrEP was first approved for use by the US Food and Drug Administration (FDA) [24]. Email notifications were configured for Ovid to alert about new records uploaded to the database from the initial search in June 2022 until the end of February 2024. Additionally, PubMed was periodically searched with a final search conducted at the end of February 2024.

### Inclusion and Exclusion Criteria

As this review focused on real-world evidence on PrEP delivery, only research that reported empirical evidence was included. Studies that reported on the different stages of the PrEP cascade, i.e. on the following outcomes: PrEP initiation (or uptake—defined as an individual starting on PrEP), PrEP persistence (or retention, continuation—defined as the duration an individual continues to take PrEP as prescribed, without interruption), PrEP adherence (which can be measured through various methods, including pharmacological measures of drug levels in the body, pill counts, or self-reported adherence over a reference period, as defined by the authors of each study), and PrEP re-initiation following discontinuation; and the facilitators and barriers to the PrEP cascade outcomes were included. Studies that presented perspectives from both the supply side (i.e. PrEP providers) and demand side (i.e. AGYW) were included. Any studies on clinical trials, or those that only reported on hypothetical willingness or acceptability of PrEP were excluded from this review. Studies that did not have age-disaggregated data for 10–24-year olds were also excluded.

### Data Extraction and Synthesis

Articles were screened using EPPI Reviewer [25]. The first author screened all title and abstracts to remove irrelevant search results using pre-specified screening questions to determine if they met the inclusion criteria. Full texts of the selected studies were then retrieved and screened for inclusion. A second reviewer (DJD) independently screened a random selection of 10% of title and abstracts, and then 10% of studies included for full-text screening to ensure accuracy. Interrater reliability was assessed using two measures: Cohen’s Kappa coefficient (0.77), which indicates substantial agreement beyond chance, and the proportion of studies with consistencies between raters (94%), reflecting excellent agreement. Any inconsistencies (6% of studies) were reconciliated through discussion and consensus.

**Fig. 1:**
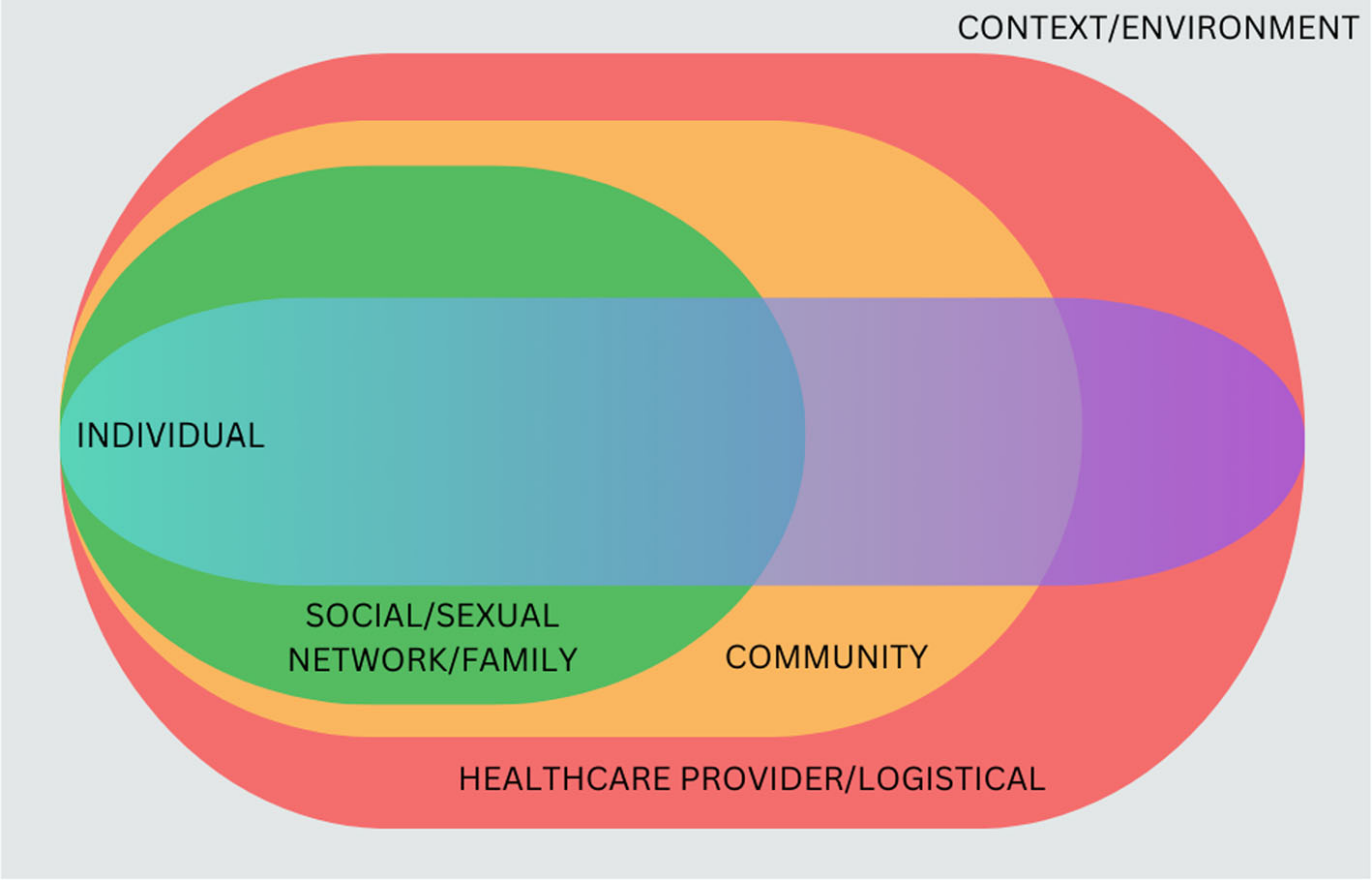
Adapted socioecological framework for HIV prevention among adolescents (adapted from [26])

Data were systematically extracted using a pre-developed data extraction sheet. Quantitative and qualitative syntheses were undertaken separately (also including data from mixed methods studies in both streams). Evidence derived from both syntheses was then merged using an adapted socioecological framework specifically tailored for HIV prevention among adolescents. This framework captures the dynamic and multifaceted nature of individual experiences, recognising that influences on adolescents are not static but are instead complex, fluid, and shaped by their interactions across individual, social/sexual network/family, community, and healthcare provider/logistical-level factors (see Fig. 1) [26]. Recommendations from AGYW and PrEP implementors were also extracted and synthesised.

During the review, it was found that the terms PrEP ‘persistence’, ‘continuation’, and ‘retention’ were used interchangeably across different quantitative studies, and their measurements varied from one study to another. We have used the term persistence in this review, but this also includes reports of continuation and retention. More salient findings on facilitators and barriers (those that were reported by three or more studies) are presented in the results section, but all findings can be found in the mapping tables (see Tables 2, 3). When findings were exclusively qualitative or quantitative, this was indicated in the results section. Otherwise, findings were synthesised and merged from both types of data.

**Table 2:** Facilitators of PrEP uptake and utilisation among AGYW in SSA identified from studies synthesised by socioecological level

Socioecological level	Facilitator	Qualitative references	Country	Quantitative references	Country
**Individual-level Facilitators**	AGYW knowledge and awareness of PrEP and its benefits	Rousseau et al. [53]	South Africa	Joseph Davey et al. [30]	South Africa
		Mudzingwa et al. [54]	South Africa		
		Jackson-Gibson et al. [55]	Kenya		
		Rogers et al. [56]	Kenya		
		Kawuma et al. [57]	Uganda		
		de Vos et al. [58]	South Africa		
		Barnighausen et al. [59]	Eswatini		
	Understanding personal risk for HIV acquisition, and increased HIV-related knowledge	Joshi et al. [60]	Uganda	Truong et al. [45]	Kenya
		Mudzingwa et al. [61]	South Africa	Martin et al. [46]	South Africa
		Rousseau et al. [53]	South Africa	Rao et al. [43]	South Africa
		Jackson-Gibson et al. [55]	Kenya	Khadka et al. [40]	South Africa
		de Vos et al. [58]	South Africa		
		Bhattacharjee et al. [50]	Kenya		
		Ngure et al. [62]	Kenya		
	Self-efficacy and internal motivation—PrEP use fostering sense of agency and self-care	Jani et al. [63]	Tanzania	Pintye et al. [42]	Kenya
		Joshi et al. [60]	Uganda	Bonner et al. [39]	Kenya
		Rousseau et al. [53]	South Africa		
		Barnighausen et al. [59]	Eswatini		
	Motherhood and pregnancy- desire to protect their child's future and maintain their own health for their children's sake	Rogers et al. [56]	Kenya	Khadka et al. [40]	South Africa
		Pintye et al. [65]	Kenya		
		Joseph Davey et al. [67]	South Africa		
		Skovdal et al. [66]	Zimbabwe		
	PrEP fosters a sense of agency and self-care – contributing to positive shifts in relationship dynamics and behaviour	Barnighausen et al. [59]	Eswatini		
		Jani et al. [63]	Tanzania		
		Joshi et al. [60]	Uganda		
		Rousseau et al. [72]	South Africa		
	Autonomy in healthcare decision-making and ability to take PrEP discreetly	Rousseau et al. [53]	South Africa		
		Wyatt et al. [64]	South Africa		
		Jackson-Gibson et al. [55]	Kenya		
		Pintye et al. [65]	Kenya		
		Barnighausen et al. [59]	Eswatini		
**Social/Sexual Network/Family-level Facilitators**	Positive response to disclosure of PrEP use within their social, sexual and family networks	Joshi et al. [60]	Uganda	Zia et al. [44]	Kenya
		Mudzingwa et al. [61]	South Africa		
		Rousseau et al. [53]	South Africa		
		Mudzingwa et al. [54]	South Africa		
		Katz et al. [68]	Kenya, South Africa		
	Ability to have transparent communication with partners regarding PrEP use—importance of partner education, and continued communication with partners regarding PrEP use	Wyatt et al. [64]	South Africa	Bonner et al. [39]	Kenya
		Jackson-Gibson et al. [55]	Kenya	Tapsoba et al. [37]	Kenya
	Parental guidance and support in sexual and reproductive health decisions—providing informational and instrumental support	Ndimande-Khoza et al. [72]	South Africa	Truong et al. [45]	Kenya
		Wong et al. [71]	Zambia	Joseph Davey et al. [30]	South Africa
	Ongoing family and social support networks help them to cope with challenges—including tangible support in the form of encouragement and reminders	Beesham et al. [69]	South Africa	Truong et al. [45]	Kenya
		Ndimande-Khoza et al. [70]	Kenya, South Africa	Joseph Davey et al. [30]	South Africa
		Pintye et al. [65]	Kenya	Mudau et al. [33]	South Africa
		Chimbindi et al. [49]	South Africa	Zia et al. [44]	Kenya
		Barnabee et al. [52]	Namibia		
		de Vos et al. [58]	South Africa		
**Community-level Facilitators**	Positive community attitudes towards PrEP—importance of engagement with stakeholders	Jackson-Gibson et al. [55]	Kenya	Joseph Davey et al. [30]	South Africa
				Tapsoba et al. [37]	Kenya
	Community-based outreach and education programmes led by communities themselves	Chimbindi et al. [49]	South Africa	Mayanja et al. [32]	Uganda
				Butler et al. [47]	South Africa
				Tapsoba [37]	Kenya
	Provision of safe spaces for education and engagement for PrEP	Jackson-Gibson et al. [55]	Kenya		
	Participation in peer support groups	Cassidy et al. [48]	South Africa	Joseph Davey et al. [30]	South Africa
	Peer-led outreach approaches	Bhattacharjee et al. [50]	Kenya		
		Chimbindi et al. [49]	South Africa		
	Peers who continue to use PrEP			Zia et al. [44]	Kenya
	Effective education campaigns informing AGYW of PrEP benefits	Cassidy et al. [48]	South Africa		
	Integration of PrEP education at antenatal care clinics	Joseph Davey et al. [67]	South Africa		
** Healthcare Provider-level and Structural Facilitators**	Adolescent-friendly, non-judgemental, and supportive counselling that provides comprehensive information, address concerns, and prepare AGYW for potential challenges	Beesham et al. [69]	South Africa	Martin et al. [46]	South Africa
		Joseph Davey et al. [67]	South Africa	Zia et al. [44]	Kenya
		Katz et al. [68]	Kenya, South Africa		
		Ngure et al. [62]	Kenya		
		Bhattacharjee et al. [50]	Kenya		
		Rousseau et al. [72]	South Africa		
	Friendly and welcoming clinic staff who offer support, information, and reassurance about PrEP	Beesham et al. [69]	South Africa		
		Rousseau et al. [72]	South Africa		
		Vera et al. [73]	Kenya		
	Logistical facilitators including integration of PrEP into existing health services, support for accessing clinics for PrEP refills, free transportation services, reduced stigma associated with PrEP dispensing	Rogers et al. [56]	Kenya	Khadka et al. [40]	South Africa
		Rousseau et al. [53]	South Africa		
		Cassidy et al. [48]	South Africa		
		Chimbindi et al. [49]	South Africa		
		Bhattacharjee et al. [50]	Kenya		
		Vera et al. [73]	Kenya		
	Consistent supply and access to PrEP at clinics			Mayanja et al. [32]	Uganda
				Rao et al. [43]	South Africa
				Butler et al. [47]	South Africa
				Zia et al. [44]	Kenya
	Adequate training for healthcare providers, coupled with non-judgmental and supportive services			Butler et al. [47]	South Africa
				Zia et al. [44]	Kenya
	Provision of affordable or complimentary PrEP services to alleviate financial barriers			Mudau et al. [33]	South Africa
				Zia et al. [44]	Kenya
	Effective programme monitoring	Bhattacharjee et al. [50]	Kenya		
	Community and hybrid service delivery models	Barnabee et al. [52]	Namibia		
	Pharmacy delivery of PrEP—enhances accessibility and convenience	Vera et al. [73]			Kenya
	Flexible clinic hours and appointment scheduling			Mayanja et al. [32]	Uganda

## Results

**Fig. 2:**
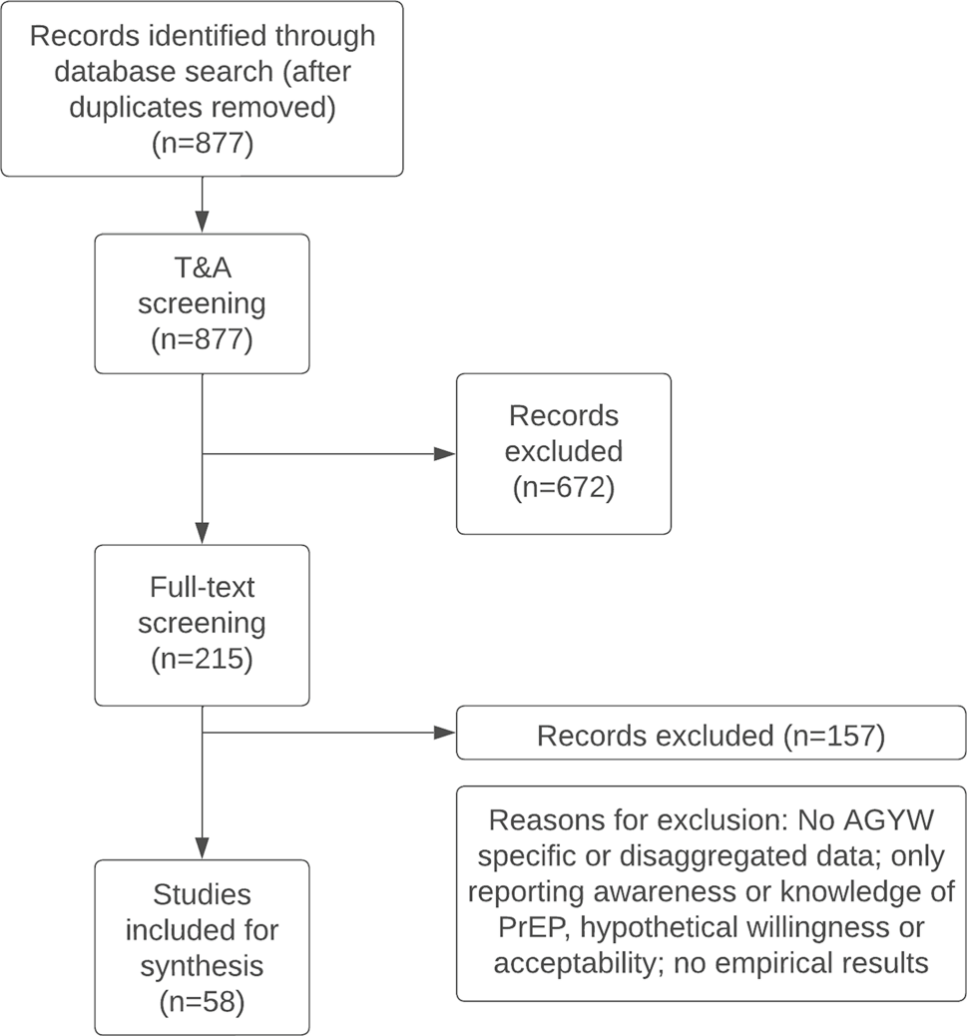
PRISMA flowchart for article selection. T&A screening = title and abstract screening

Out of 877 studies identified following database search and deduplication, 58 studies were eligible for inclusion (see Fig. 2 for the PRISMA flowchart). More than half (55%) of the studies used qualitative methods, 38% used quantitative, and 7% used mixed methods. Most studies were conducted in South Africa (44%) and Kenya (38%), with the rest conducted in Uganda (6%), Zimbabwe (4%), Eswatini (3%), Zambia (1%), Namibia (1%), and Tanzania (1%). Only studies on oral PrEP met the inclusion criteria for real-world delivery; studies on other PrEP modalities including injectable PrEP did not meet the inclusion criteria due to the novelty of these methods.

**Table 3:** Barriers of PrEP uptake and utilisation among AGYW identified from studies synthesised by socioecological level

Socioecological level	Barriers	Qualitative references	Country	Quantitative references	Country
**Individual-level Barriers**	Stigma and misconceptions among families, peers, communities—leading to challenges including the need to conceal PrEP use	Beesham et al. [74]	South Africa	Zia et al. [44]	Kenya
		Wagner et al. [75]	Kenya	Mayanja et al. [32]	Uganda
		Mudzingwa et al. [61]	South Africa	Tapsoba et al. [37]	Kenya
		Rousseau et al. [72]	South Africa		
		Wyatt et al. [64]	South Africa		
		Escudero et al. [76]	Kenya		
		Skovdal et al. [78]	Zimbabwe		
		Beesham et al. [69]	South Africa		
		Jani et al. [63]	Tanzania		
		Joshi et al. [60]	Uganda		
		Perry et al. [77]	Kenya		
		Vera et al. [73]	Kenya		
		Ndimande-Khoza et al. [70]	Kenya, South Africa		
		de Vos et al. [58]	South Africa		
		Cassidy et al. [48]	South Africa		
		Chimbindi et al. [49]	South Africa		
		Bhattacharjee et al. [50]	Kenya		
		Barnabee et al. [52]	Namibia		
		Mudzingwa et al. [54]	South Africa		
	Disclosure-related factors—including discouraging and negative reactions, the need for family approval, or family prohibition	Beesham et al. [74]	South Africa		
		Mudzingwa et al. [61]	South Africa		
		Rousseau et al. [53]	South Africa		
		Skovdal et al. [84]	Zimbabwe		
		Joshi et al. [60]	Uganda		
		Perry et al. [77]	Kenya		
		Ndimande-Khoza et al. [70]	Kenya, South Africa		
	Side-effects: anticipated/fear of or actual experiences—including gastrointestinal discomfort and headaches	Beesham et al. [74]	South Africa	Celum et al. [28]	Kenya, South Africa
		Duby et al. [79]	South Africa	Mudau et al. [33]	South Africa
		Jackson-Gibson et al. [55]	Kenya	Bonner et al. [39]	South Africa
		Rogers et al. [56]	Kenya	Pintye et al. [42]	Kenya
		Rousseau et al. [72]	South Africa		
		Wyatt et al. [64]	South Africa		
		Escudero et al. [76]	Kenya		
		de Vos et al. [58]	South Africa		
		Chimbindi et al. [49]	South Africa		
		Barnabee et al. [52]	Namibia		
		Mudzingwa et al. [54]	South Africa		
	Pill fatigue/burden, and other factors related to pill-taking such as the size of the pill	Beesham et al. [74]	South Africa	Bonner et al. [39]	South Africa
		Perry et al. [77]	Kenya	Pintye et al. [42]	Kenya
		Rousseau et al. [72]	South Africa		
		Duby et al. [79]	Africa		
		Bjertrup et al. [80]	Eswatini		
		de Vos et al. [58]	South Africa		
		Kawuma et al. [57]	Uganda		
	Pregnancy and breastfeeding-related fears of harmful effects on their foetus or infants	Joshi et al. [60]	Uganda		
		Wyatt et al. [64]	South Africa		
		Rogers et al. [56]	Kenya		
		Pintye et al. [65]	Kenya		
	Postpartum period—change in routine and difficulties due to motherhood	Wyatt et al. [64] Beesham et al. [74]	South Africa		South Africa
		Pintye et al. [65]	Kenya		
	Lack of understanding, misconceptions and misinformation fuelled by inaccurate online/social media sources	Duby et al. [79]	South Africa	Kinuthia et al. [31]	Kenya
		Rousseau et al. [53]	South Africa	Giovenco et al. [29]	South Africa, Zimbabwe
		Escudero et al. [76]	Kenya		
		de Vos et al. [58]	South Africa		
		Perry et al. [77]	Kenya		
	Low or lowered perception of HIV risk	Mudzingwa et al. [61]	South Africa	Haberer et al. [51]	Kenya
		Perry et al. [77]	Kenya	Sila et al. [36]	Kenya
		Rousseau et al. [72]	South Africa	Martin et al. [46]	South Africa
		de Vos et al. [58]	South Africa	Ogolla et al. [41]	Kenya
	Mobile lifestyles or frequent relocation, and being away from home when PrEP needed to be taken lead to forgetfulness or changes in routine which triggered missed doses	Beesham et al. [74]	South Africa		
		Beesham et al. [69]	South Africa		
		Jackson-Gibson et al. [55]	Kenya		
		Pintye et al. [65]	Kenya		
		de Vos et al. [58]	South Africa		
		Chimbindi et al. [49]	South Africa		
		Wyatt et al. [64]	South Africa		
	Other life priorities, scheduling conflicts, and difficulties in accessing PrEP	Beesham et al. [74]	South Africa		
		Mudzingwa et al. [61]	South Africa		
		Perry et al. [77]	Kenya		
		de Vos et al. [58]	South Africa		
		Rousseau et al. [72]	South Africa		
	Clinic-related anxiety due to negative experiences with healthcare providers, and concerns around being judged at clinics. Clinics perceived as not youth-friendly and overworked	Escudero et al. [76]	Kenya		
		Omollo et al. [85]	Kenya		
	Decreased motivation and/or capacity to engage with PrEP services due to experiencing depressive symptoms and intimate partner violence			Bonner et al. [39]	South Africa
				Martin et al. [46]	South Africa
				Heck et al. [38]	Kenya
	Lack of internal motivation to take PrEP	Rousseau et al. [72]	South Africa		
		Pintye et al. [65]	Kenya		
	Reluctance to retest for HIV due to emotional stress and logistical barriers	Escudero et al. [76]	Kenya		
**Social/Sexual Network/Family-level Barriers**	Partner influence—negative reactions from male partners, viewing PrEP use as sign of mistrust or infidelity leading to relationship discord, and partners sharing inaccurate information about PrEP	Bjertrup et al. [80]	Eswatini	Butler et al. [47]	South Africa
		Jani et al. [63]	Tanzania	Pintye et al. [42]	Kenya
		Katz et al. [68]	Kenya, South Africa		
		Rogers et al. [56]	Kenya		
		Skovdal et al. [78]	Zimbabwe		
		de Vos et al. [58]	South Africa		
		Pintye et al. [65]	Kenya		
	Fear of partner—partner prohibitions/disapproval or anticipated conflict caused by PrEP use, experiences of intimate partner violence (IPV)	Barnighausen et al. [59]	Eswatini	Ohiomoba et al. [35]	Kenya
		Holmes et al. [81]	South Africa	Rao et al. [43]	South Africa
		Mudzingwa et al. [61]	South Africa		
		Perry et al. [77]	Kenya		
		Rousseau et al. [72]	South Africa		
	Parental resistance and family disapproval, misinformation, and parents feeling poorly consulted or informed	Bjertrup et al. [80]	Eswatini	Joseph Davey et al. [30]	South Africa
		Duby et al. [79]	South Africa	Ohiomoba et al. [35]	Kenya
		Bhattacharjee et al. [50]	Kenya		
		Ndimande-Khoza et al. [70]	Kenya, South Africa	Truong et al. [45]	Kenya
	Stigma associated with HIV and PrEP usage within social environments, and negative social influences or absence of supportive networks	Duby et al. [79]	South Africa	Zia et al. [44]	Kenya
**Community-level Barriers**	Community stigma and misconceptions	Duby et al. [79]	South Africa	Butler et al. [47]	South Africa
		Jackson-Gibson et al. [55]	Kenya	Tapsoba et al. [37]	Kenya
		Lanham et al. [82]	Kenya		
		Wong et al. [71]	Zambia		
	Resistance from religious leaders	Duby et al. [79]	South Africa		
		Katz et al. [68]	Kenya, South Africa		
		Perry et al. [77]	Kenya		
	Patriarchal and social norms	Skovdal et al. [84]	Zimbabwe		
Healthcare Provider-level and Structural Barriers**Healthcare Provider-level and Structural Barriers**	Resource allocation and perceived workload and burden of incorporating PrEP services into existing workflows	Barnighausen et al. [59]	Eswatini		
		Jackson-Gibson et al. [55]	Kenya		
		Skovdal et al. [84]	Zimbabwe		
		Chimbindi et al. [49]	South Africa		
		Bhattacharjee et al. [50]	Kenya		
		Barnabee et al. [52]	Namibia		
		O'Malley et al. [83]	Kenya, South Africa		
	Lack of training and knowledge among HCPs—concerns among HCPs about PrEP's safety and efficacy, particularly during pregnancy and breastfeeding, and drug resistance and sexual behaviours of AGYW which leads to reluctance to provide PrEP services	O'Malley et al. [83]	Kenya, South Africa	Bonner et al. [39]	South Africa
		Chimbindi et al. [49]	South Africa	Mudau et al. [33]	South Africa
		Perry et al. [77]	Kenya		
	Logistical barriers—particularly in rural areas due to distance from services, protracted waiting times, screening requirements, and restricted clinic hours	Beesham et al. [69]	South Africa	Martin et al. [46]	South Africa
		Joshi et al. [60]	Uganda	Mayanja et al. [32]	Uganda
		Pintye et al. [65]	Kenya	Butler et al. [47]	South Africa
		de Vos et al. [58]	South Africa	Rao et al. [43]	South Africa
		Vera et al. [73]	Kenya		
	Service disruptions due to health facility closures, inadequate stock management and stockouts, and inadequate follow-up and support from HCPs, as well as disruptions caused by COVID-19 which exacerbated these challenges	Beesham et al. [69]	South Africa	Ogolla et al. [41]	Kenya
		Duby et al. [79]	South Africa	Rao et al. [43]	South Africa
				Mayanja et al. [32]	Uganda
				Butler et al. [47]	South Africa
				Khadka et al. [40]	South Africa
		Beesham et al. [74]			South Africa
	Financial constraints and absence of affordable PrEP options			Ogolla et al. [41]	Kenya
				Rao et al. [43]	South Africa

### Quantitative Findings on PrEP Initiation, Persistence, and Adherence

The synthesis of data from n = 22 quantitative studies and n = 3 mixed methods studies conducted in South Africa, Kenya, Zimbabwe and Uganda showed large variations in proportions of PrEP initiation among eligible HIV-negative AGYW, ranging from 2 to 82% (measured by 24 studies) [27–50]. But it was clear across the studies that PrEP persistence decreased when measured across time, e.g. 3-months, 6-months and/or 12-months, with persistence rates ranging from 29 to 58% after 3-months (measured by 17 studies) [28, 30–35, 37, 39–43, 48, 50–52]. Only three studies measured PrEP adherence, with the majority (67%) of participants having consistently low adherence [48], adherence declining over time and demonstrably low adherence when measured at month 6 (18%) [29], and month 22 (15%) [51].

### Merged Qualitative and Quantitative Findings

#### Facilitators for PrEP Uptake and Utilisation

##### Individual-Level Facilitators

Qualitative findings from studies in four SSA countries revealed that a positive perception of PrEP as an effective HIV prevention method significantly motivated AGYW to initiate and adhere to PrEP [53–59]. Quantitative data indicated that adequate knowledge and awareness about PrEP encouraged initiation [30].

Findings from studies in Kenya, South Africa, and Uganda showed that AGYW's understanding of their personal risk for HIV acquisition was a crucial motivator for PrEP uptake. Qualitative and quantitative studies emphasised that recognising their greater likelihood of exposure to HIV drove AGYW to use PrEP [40, 43, 45, 46, 53, 55, 58, 60–62]. Additionally, the persistent perception of HIV risk motivated AGYW to continue using PrEP [43, 46], or to re-initiate [45, 46].

Findings from studies in five SSA countries underscored the significant role of self-efficacy and internal motivation. Qualitative findings from studies in Eswatini, Kenya, and South Africa highlighted that PrEP use fostered a sense of agency and self-care and contributed to a sense of relief and hope for the future, as well as positive shifts in relationship dynamics and risk behaviour [53, 59, 60, 63]. The importance of AGYW's autonomy in healthcare decision-making and their ability to take PrEP discreetly was essential [53, 55, 59, 64, 65]. Quantitative data from Kenya confirmed that higher levels of self-efficacy and internal motivation were associated with increased PrEP initiation and persistence [39, 42].

Motherhood or pregnancy emerged as strong motivators for PrEP use in qualitative studies from Kenya, South Africa, and Zimbabwe. The desire to protect their child's future and maintain their own health for their children's sake drove AGYW to initiate and adhere to PrEP [40, 56, 65–67].

##### Social/Sexual Network/Family-Level Facilitators

The synthesis of qualitative and quantitative findings underscored the pivotal role of positive social, sexual, and family networks in facilitating effective PrEP uptake, persistence and adherence among AGYW. Qualitative data from Kenya and South Africa noted that positive responses to PrEP use disclosure within these networks significantly empowered AGYW to make informed decisions about their health and well-being, and use PrEP [53, 54, 60, 61, 68]. Quantitative findings from a study in Kenya supported this, indicating that support from peers, partners, or healthcare providers played a role in encouraging individuals to re-engage and resume PrEP use [44].

Qualitative studies from South Africa and Kenya highlighted the importance of educating male sexual partners about PrEP, which not only encouraged their partners' participation but also strengthened relationship dynamics [55, 64]. Quantitative data showed that continued communication with partners regarding PrEP use enhanced persistence among AGYW [37, 39].

Ongoing family and social support and approval, including tangible support such as encouragement and reminders, also played a role in facilitating PrEP initiation and maintenance by fostering a supportive environment for PrEP adherence and persistence [49, 52, 58, 65, 69, 70]. Qualitative findings from studies in Zambia and South Africa emphasised the role of parental guidance and support in daughters' SRH decisions, particularly in providing informational and instrumental support such as guidance on dealing with side effects and accompaniment to clinics [30, 45, 70, 71].

##### Community-Level Facilitators

Findings from studies in Kenya, South Africa, and Uganda highlighted the significant role of positive community attitudes towards PrEP, which significantly mitigated stigma and enhanced uptake among AGYW. The engagement of community stakeholders helped address misconceptions and resistance and promoted a positive perception of PrEP within the community for AGYW [30, 37, 55]. For example, a study in Kenya showed engagement and collaborations with the Kenyan Ministry of Health and key opinion leaders was important in overcoming initial barriers to PrEP implementation and ensuring successful PrEP delivery to AGYW [55].

##### Healthcare Provider-Level and Structural Facilitators

The successful uptake and continued use of PrEP by AGYW was significantly influenced by several healthcare provider-level and logistical facilitators. Qualitative findings from studies in Kenya and South Africa emphasised the importance of adolescent-friendly, non-judgmental, and supportive counselling, including counselling services integrated into Antenatal Care (ANC) and Postnatal Care (PNC) services. Counsellors played a role in facilitating PrEP acceptance by providing comprehensive information, addressing concerns, and preparing AGYW for potential challenges associated with PrEP use [44, 46, 50, 62, 67–69, 72].

Studies from South Africa and Kenya reported how the attitude and demeanour of healthcare providers also significantly impacted PrEP uptake. Friendly and welcoming clinic staff who offered support, information, and reassurance regarding PrEP and its potential side effects helped alleviate AGYW’s concerns and promoted continued engagement with PrEP services [69, 72, 73]. Additional logistical support for access to clinics for PrEP refills such as free transportation services, reduced stigma associated with PrEP dispensing, and integration of PrEP into existing healthcare systems enhanced convenience and accessibility and ensured AGYW could easily initiate and persist on PrEP services [40, 48–50, 56, 72, 73].

#### Barriers to PrEP Uptake and Utilisation

##### Individual-Level Barriers

The synthesis of qualitative and quantitative findings identified several significant individual-level barriers to the uptake and consistent use of PrEP among AGYW. The most salient barrier reported by studies in five SSA countries was anticipated or experienced stigma and misconceptions about PrEP among families, peers, and communities of AGYW, including confusion of PrEP with antiretroviral therapy (ART) for HIV treatment. This created considerable challenges for PrEP persistence and adherence due to the reported need to conceal PrEP use, which exacerbated challenges with keeping PrEP schedules and missing doses, and eventually lead to discontinuation [48–50, 52, 54, 58, 60, 61, 63, 64, 66, 69, 70, 72–77]. Issues related to disclosure of PrEP use, such as discouraging and negative reactions, the need for family or partner approval, and family prohibition reported in studies from four SSA countries also made consistent PrEP use challenging [53, 60, 61, 70, 74, 77, 78]. Lack of knowledge and misconceptions about PrEP, worsened by misinformation from online sources, such as negative rumours about PrEP's safety and side effects, was reported in studies from Kenya, South Africa and Zimbabwe, and contributed to AGYW's resistance to PrEP uptake and discontinuation [29, 31, 53, 58, 76, 77, 79].

Fear and the actual experience of side effects, including gastrointestinal discomfort and headaches, were well documented in studies from South Africa and Kenya, and acted as a deterrent for PrEP initiation and retention [28, 33, 39, 42, 49, 52–56, 58, 64, 74, 76, 79]. Furthermore, issues related to pill fatigue, pill burden, and the physical size of the pill which made swallowing the pill difficult also discouraged AGYW from consistent PrEP use in studies from four SSA countries [39, 42, 53, 57, 58, 74, 77, 79, 80]. Pregnant and breastfeeding AGYW in studies from Kenya, South Africa and Uganda also feared any harmful effects PrEP might have on their foetus or infants, or future fertility, and cited higher percentage of gastrointestinal side effects, which negatively impacted their uptake and use of PrEP [40, 56, 60, 64, 65].

Furthermore, low perceived risk of HIV acquisition emerged as a significant barrier in studies from South Africa and Kenya, including qualitative studies which illustrated how changes in relationship status or condom use contributed to a diminished perception of HIV risk, and led to PrEP discontinuation [36, 41, 46, 51, 53, 58, 61, 77]. Additionally, mobile lifestyles, frequent relocations, and being away from home interfered with a consistent PrEP regimen [49, 55, 58, 64, 65, 69, 74]. Other life priorities (such as work or school), scheduling conflicts, and difficulties in accessing PrEP distribution sites also impeded consistent use [53, 58, 61, 74, 77]. AGYW with mental health issues, which was often associated with stigma, discrimination, social isolation, and intimate partner violence (IPV), faced challenges in persistent PrEP use [38, 39, 46].

##### Social/Sexual Network/Family-Level Barriers

Quantitative and qualitative evidence from studies in five SSA countries underscored the highly influential role of male partners in AGYW's PrEP decisions. Negative reactions, opposition, perception of PrEP as a sign of mistrust or infidelity, lack of support, or inaccurate information about PrEP from male partners significantly undermined AGYW's motivation to initiate or persist in PrEP use [42, 47, 56, 58, 63, 65, 66, 68, 80]. In addition, experiences of IPV, fear of partners, and concerns about relationship instability further hindered PrEP uptake and persistence [35, 43, 59, 61, 72, 77, 81].

Studies in Eswatini, Kenya, and South Africa showed parental opposition, fuelled by misconceptions about PrEP (such as mistaking it for ART or abortion pills), complicated AGYW's access to accurate information and support, subsequently negatively impacted PrEP uptake [30, 35, 45, 50, 70, 79, 80].

##### Community-Level Barriers

Studies in four SSA countries emphasised that community stigma, misconceptions, and disapproval—often fuelled by a lack of awareness and the incorrect belief that PrEP promotes risky sexual behaviour—created a hostile environment and significantly hindered PrEP uptake and use [37, 47, 55, 71, 79, 82]. Religious opposition, rooted in the perception that PrEP promotion conflicted with religious values, and patriarchal norms and beliefs in conservative communities reinforced negative community perceptions around PrEP and created additional barriers [68, 77–79].

##### Healthcare Provider-Level and Structural Barriers

Studies from five SSA countries highlighted the challenges related to resource allocation and the perceived workload and burden by healthcare providers of incorporating PrEP services into existing workflows. Qualitative findings indicated that limited financial and human resources restricted the time healthcare providers could spend with AGYW, impacting the timely availability of PrEP services and counselling, which consequently reduced AGYW’s motivation to continue PrEP [49, 50, 52, 55, 59, 78, 83]. A lack of training and knowledge or negative attitudes among healthcare providers, concerns about PrEP's safety and efficacy, particularly during pregnancy and breastfeeding, and reluctance to provide PrEP services due to worries about drug resistance and sexual behaviours of AGYW hindered PrEP initiations [33, 39, 49, 77, 83].

Studies from Kenya, South Africa and Uganda highlighted structural issues such as limited and unreliable transport, financial difficulties, long waiting times at clinics, restricted clinic opening hours, and screening requirements as deterrents for PrEP uptake and access among AGYW [32, 43, 46, 47, 58, 60, 65, 69, 73]. Inadequate stock management and frequent stockouts at healthcare facilities limited the availability of PrEP and impeded initiation [32, 40, 41, 43, 47, 69, 74, 79]. Postpartum women who no longer attended ANCs faced additional difficulties accessing PrEP appointments or picking up refills [64, 65, 74].

#### AGYW and PrEP Provider Recommendations for PrEP Delivery from Qualitative Studies

PrEP providers and AGYW in qualitative studies recommended various strategies to improve PrEP uptake, adherence, and persistence among AGYW. Both PrEP providers and AGYW emphasised the importance of community leader involvement for sensitisation, promotion, and implementation of PrEP initiatives, alongside reaching AGYW within educational settings to disseminate PrEP knowledge [59, 84]. Collaboration between community-based implementers and clinic staff was highlighted as crucial for promoting PrEP uptake through referrals [79]. Raising awareness about PrEP through community campaigns and discussions was recommended to address stigma and normalise its usage, alongside dispelling misconceptions about PrEP’s association with increased sexual activity and promiscuity [79, 82–84]. AGYW specifically suggested integrating PrEP delivery into existing healthcare services such as ANCs and informing their social networks about PrEP benefits to reduce stigma and misconceptions [67–69]. They also emphasised on the need for PrEP advocacy through peers, recommended couple PrEP counselling and comprehensive PrEP education for male partners, and improved parent–child communication on SRH topics to foster mutual support and understanding [53, 54, 63, 71, 77].

Parental engagement and fostering open communication and support through trustful relationships was considered crucial by both AGYW and PrEP providers for supporting AGYW’s effective use of PrEP [79, 82]. Encouraging couples’ HIV testing and counselling was also recommended to enhance PrEP uptake [82]. Intensified counselling, tailored to AGYW’s unique circumstances and needs, was suggested to address adherence and persistence challenges, with a conversational approach during PrEP counselling appreciated by AGYW [82, 83, 85]. Improving patient-centred care, ensuring privacy, and providing non-judgmental treatment are key components of youth-friendly services. This also requires sensitisation and training programmes for healthcare workers, community health workers, and peer educators. [76, 83–85]. Offering discreet options for administering PrEP could address concerns about stigma, while tangible support through tracking missed client visits and providing follow-up through calls, SMS, and home visits could promote persistence [82, 84]. AGYW also suggested ensuring suitable clinic hours, integrating queues to pick up PrEP at health facilities, and providing reliable transportation assistance [69, 77]. Discreet administration options and expanding PrEP service locations to community settings and schools were suggested to address disclosure and storage issues [53, 69, 72].

Leveraging social media platforms was recommended by both AGYW and PrEP providers to facilitate reaching AGYW with PrEP-related information, encouraging them to reflect on their HIV risk and empowering them to take charge of their health [82, 85]. Utilising peer educators and implementing demand creation activities could increase PrEP awareness [84]. Reimbursing transport costs and exploring alternative delivery points beyond HIV treatment clinics could improve accessibility to PrEP services, while involving key opinion leaders, including adolescents, in policymaking could ensure effective and sustainable PrEP implementation [76].

## Discussion

We synthesised evidence on PrEP delivery to AGYW in SSA in this review, highlighting multifaceted challenges in real-world PrEP delivery and underscoring poor PrEP uptake and persistence due to barriers at various socioecological levels. The geographic focus on South Africa and Kenya emphasises the need for more comprehensive studies across SSA. Variability in PrEP initiation, influenced by factors such as location and HIV prevalence, underpins the necessity for localised and context-specific strategies [86, 87].

Significant gaps exist at each stage of the PrEP cascade – stigma, misinformation, and structural challenges hinder PrEP awareness, access, and initiation. Social support, healthcare provider interactions, and structural barriers affect PrEP adherence and persistence. There is a notable lack of strategies that support AGYW in long-term persistence and keeping them engaged in PrEP services. Interventions should address specific barriers and facilitators for AGYW at each stage of the PrEP cascade. This includes understanding the role of continuous PrEP education and engagement with AGYW, their social networks, and their communities for effective implementation. Differentiated Service Delivery (DSD) models have shown effectiveness for ART delivery and retention, such as through adherence clubs and community ART groups – these strategies could also be adopted for PrEP services to address similar issues in adherence [88, 89].

The need for improved PrEP delivery mechanisms and addressing accessibility issues surrounding costs and distance to clinics suggest the necessity for policy interventions. Alternative PrEP delivery mechanisms, such as mobile clinics and pharmacies, nurse-led models, and community-based approaches, should be explored to promote equitable access. These strategies have already shown effectiveness in other populations, and in contraceptive delivery. For example, a systematic review on PrEP uptake among female sex workers in SSA found community-based models, including pharmacies, significantly improved retention rates [90]. Trained community health workers showed success in the delivery of injectable contraceptives in SSA communities [91]. Peer- and community-led HIV responses also demonstrated improvements in HIV service access, utilisation, linkage, retention, and quality [92].

Innovative, people-focused approaches are essential to improving PrEP access and adherence. These might include virtual consultations and support systems for adherence, mobile health units to reach remote populations, and the use of community-based PrEP ambassadors [93]. Research from Kenya has indicated that AI-driven telehealth platforms could significantly alleviate the pressure on healthcare providers and systems in resource-limited settings [94]. Furthermore, more practical solutions to help AGYW conceal PrEP and navigate stigma is needed, such as changing the design of the pills or the containers to prevent confusion with ART [84, 95, 96].

This review also emphasises the importance of community-centric approaches – to involve not only healthcare providers but also community leaders, parents, and peers in promoting and normalising PrEP use. Social, religious, and cultural barriers continue to create significant barriers to PrEP uptake, especially since cultural and religious norms that oppose the use of preventive measures such as PrEP may deter AGYW from seeking care [68, 78, 79]. In some contexts, PrEP use may be linked to promiscuity, conflicting with gender or religious norms [78]. These perceptions can cause AGYW to hesitate in seeking SRH services and PrEP, due to fear of social exclusion or familial rejection. To overcome this, interventions must be culturally sensitive, involving community and religious leaders and incorporating relevant education into HIV prevention programmes to foster support for PrEP and improve SRH outcomes for AGYW. For example, community-supported models of care, such as for ART delivery and condom distribution, have shown effectiveness in reducing HIV stigma and increasing condom use [97].

Pregnancy and motherhood also emerged as strong motivators for PrEP use, with AGYW driven by the desire to protect their children's future and maintain their own health [98–100]. This highlights the need for more research focusing on AGYW mothers, as understanding their unique motivations and barriers can inform the design of tailored interventions that address the specific needs of this population with additional vulnerabilities. Integrating services in family planning (FP), SRH services, and ANCs is crucial to ensure comprehensive support for AGYW and especially those who are pregnant, breastfeeding, and mothers. Such integration can enhance access to PrEP and other essential health services, contributing to better health outcomes for both mothers and their children. Integration of SRH and HIV services is recommended by the WHO, as it would improve access, quality of ANCs, and healthcare provider productivity [101]. This approach could also help reduce stigma and structural barriers for AGYW. A systematic review on integrating FP and HIV testing services also found that integrating services improved uptake, service quality, client satisfaction, whilst reducing stigma and structural barriers [102].

Incorporating recommendations from AGYW and PrEP providers is overdue; their role in shaping PrEP strategies is essential. Insights from AGYW ensure a thorough understanding of their unique needs, preferences, and challenges related to effective PrEP uptake and utilisation. Input from PrEP providers ensure practical feasibility and alignment with clinical practices, addressing not only the practical aspects of PrEP delivery but also the societal and interpersonal factors that influence its acceptance and utilisation. This collaborative approach helps develop evidence-based interventions that are also culturally sensitive.

### Strengths and Limitations

This review comprehensively captured existing evidence on real-world PrEP delivery by including both quantitative and qualitative studies. It specifically extracted data on facilitators and barriers to PrEP uptake and utilisation across the socioecological levels. However, the exclusion of grey literature due to its lack of peer review, and conference abstracts may have resulted in missing relevant data. The focus on real-world PrEP delivery meant only studies on oral PrEP were included, as studies on emerging new PrEP modalities such as injectable PrEP did not meet the inclusion criteria.

However, identified studies are valuable for informing the implementation of new PrEP modalities that are being made available, such as the vaginal ring and injectable PrEP methods. We urgently need to design tailored strategies, including tailored education, community engagement, and healthcare provider training for new PrEP modalities as they begin to roll out. Therefore, while findings in this review are rooted in oral PrEP delivery, this review provides a foundational framework for enhancing the uptake, persistence and adherence of new PrEP modalities among AGYW in SSA.

## Conclusion

In this scoping review we mapped the complexities surrounding PrEP uptake and utilisation among AGYW in SSA. We found significant barriers at each stage of the PrEP cascade, and a notable lack of strategies to support AGYW in long-term persistence and engagement with PrEP services. However, we also identified key facilitators that, if strengthened, could improve PrEP uptake and long-term engagement. Future strategies for PrEP delivery should not only focus on overcoming barriers but also on strengthening the facilitators that encourage uptake and persistence. Every day, AGYW in SSA face the risk of HIV acquisition, and there is the opportunity to prevent these new HIV cases through effective PrEP delivery and supporting effective utilisation as part of comprehensive HIV prevention packages. Policymakers, researchers, and stakeholders must act now to address the barriers to PrEP uptake and utilisation including stigma, negative sexual/family/social network influences, and structural barriers. Effective strategies for PrEP delivery need to be implemented, which could include DSD models, innovative approaches such as telehealth, and integrating PrEP with existing services. Understanding the unique needs of AGYW mothers and addressing their specific barriers can further inform tailored interventions for this particularly affected population. Long acting PrEP modalities, which could help address barriers, need to be made widely available to bring us closer to the goal of an HIV-free generation.

## Supplementary Information

Below is the link to the electronic supplementary material.

Supplementary file1 (DOCX 136 KB)
